# Some Observations Respecting the Cause, Nature, and Cure of Gout

**Published:** 1802-10-01

**Authors:** 

**Affiliations:** Surgeon, of Oxford Street


### *Some Observations respecting the Cause, Nature, and Cure of Gout; communicated by* Mr. Blegborough, *Surgeon, of Oxford Street.*

THE numerous authors, who, ſince the time of Hippocrates, have written on Gout, have differed ſo materially reſpecting its cauſe, nature, and cure, that the ſcepticiſm of the moſt eminent phyſicians of the preſent day, with reſpect to every thing that concerns the treatment of it, has almoſt ceaſed to ſurprize. Some have carried their doubts ſo far as to believe that nothing ought to be done in the diſeaſe, but that it is better to leave it altogether to time, patience, and flannel. In Germany it is not unuſual for one, who is known to have been attacked with the piles, to have the earneſt congratulations of all his neighbours, as a circumſtance in which his welfare is much concerned. In England this is in ſome meaſure the caſe with reſpect to the Gout. They are equally ridiculous. That theſe diſeaſes keep off others, I cannot believe; that they diſpoſe to many, and thoſe of the worſt kind, I have little doubt. This unwarrantable indifference in regular practitioners, to a diſeaſe for which, nevertheleſs, much may be done, has, as might be expected, opened a door to the pretenſions of the Empyric, whoſe random attempts will not ceaſe to ſcourge, till the former ſhall have it in their power to propoſe ſome more ſucceſsful mode of treatment. The cauſes and cure of Gout are ſo intimately connected, that when the latter is attempted, the former ought never for a moment to be loſt ſight of. Theſe cauſes are the improper and premature applications of thoſe powers which, when duly applied, ſuſtain life; as, heat, the ingeſta, the blood, the powers of the mind, as ſenſation, paſſion, and thought.

From the moment that life commences, every period of it demands the application of its appropriate degree of thoſe exciting powers; and the ſyſtem is in a low, high, or exhauſted ſtate of excitement, in proportion as they are applied in defect, exceſs, or in a too uniformly exceſſive degree.

Gout is generally the conſequence of the latter, or, in other words, ariſes from an unduly exhauſted excitability, which induces debility and relaxation of the ſolids, deſtroying their tone, and rendering them unfit to reſiſt the action of the vaſcular ſyſtem. Thus are the fluids puſhed beyond their proper channels, not to be returned but by an effort at the expence of the ſtrength of the whole ſyſtem. Among the cauſes, the ingeſta act moſt powerfully, for they induce moſt of the reſt, even the moſt inordinate affections of the mind. The uſe of alcohol, in whatever ſhape, is beyond doubt ſo active an agent in producing the the diſeaſe, that I am of opinion it never yet occurred to any perſon, however ſtrong his hereditary prediſpoſition, and in whatever exceſs all the other cauſes might have been applied, unleſs this one had alſo been ſuperadded. Alcohol moſt effectually debilitates the functions of the ſtomach, on which the due performance of all the other functions of the body depends. It particularly diſturbs thoſe of the liver, a torpid ſtate of which is frequently the immediate cauſe of ſevere paroxyſms of the diſeaſe. The puniſhment of Prometheus then ſcarcely exceeded his crime! High ſeaſoned food and all the other cauſes act their parts, but ſubordinate to that already mentioned. The depreſſing paſſions of the mind, as fear, grief, &c. powerfully contribute to the formation of this diſeaſe, whoſe foundation is often laid in early life, and before we are in the leaſt aware of it. The ſtate of collapſe *poſt coitum* alſo peculiarly prediſpoſes to it, and its ſevereſt paroxyſms often immediately ſucceed immoderate exertions of this nature. The Gout, except under ſome peculiar circumſtances, where the hereditary prediſpoſition is very ſtrong, and conjoined with many of the exciting cauſes, ſeldom attacks before the age of thirty-five. At this period, if the reſiſting and contractile power of the animal fibre be weakened, and the tone of it loſt by debauchery and idleneſs, the diſeaſe will make its appearance even without the hereditary prediſpoſition.

Though I intend not to deny that our conſtitutions like our perſons, are frequently very ſimilar to thoſe of our parents; yet I mean to contend, that any one, by attention to regimen, &c. may entirely alter that ſpecific ſtate of the ſolids, which conſtitutes the gouty diatheſis, however ſtrong his hereditary prediſpoſition to it may have been. That the Gout then is neither hereditary nor incurable are facts which it greatly imports the deſcendants of gouty perſons to know. The contrary idea, as erroneous as miſchievous, has, without doubt, much increaſed its martyrs, as nothing is more common than to hear a perſon declare, that as his father had the Gout, it is impoſſible for himſelf to eſcape it; ſo, why ſhould he deprive himſelf of the many gratifications which are within his reach. The conſequence of ſuch reaſoning needs no elucidation, as every day affords but too many melancholy proofs of it. The preſent Duke of Portland, and Dr. Gregory of Edinborough, who in early life were both attacked by the diſeaſe, and whoſe fathers both ſuffered ſeverely from it, are living teſtimonies that it is neither hereditary nor incurable, but that an entire and ſyſtematic change, from that mode of living which induces it, will be ſufficient to enſure the Patient from its ſubſequent attacks, and that this change is not, as has been erroneouſly imagined, incompatible with the enjoyment of good health.

Though

Though this difeafe, in general, occurs without evident external caufes, yet they are not always wanting, as bruifes, expofure to cold, &c. Pyrexia, though fometimes wanting in flight cafes, feems always to accompany violent paroxyfms of it. Stomach complaints I believe always precede it, and ought to be confidered as its never-failing harbingers. We cannot then pay too great attention to the ftate of the ftomach, as on the due performance of its functions fo much depends. Whatever overloads or heats this organ, gives rife to an excitement of the whole fyftem, greater than natural, and ultimately induces that fpecies of debility without which the difeafe cannot exift. In proportion as the ftrength of digeftion diminifhes, parts more effential to life become affected: The powers of the fyftem not being adequate to the eftablifhment of regular paroxyfms on the extremities as formerly, the lungs—head—heart, &c. begin to fuffer in turn, and we have difficulty of breathing, afthma—giddinefs, intellectual confufion—palpitations, faintings—and very often fudden death. The atonic, retrocedent, and mifplaced Gout, are diftinctions on which too much ftrefs has been placed; they ought to be confidered only as advanced ftages of the difeafe, and prove to us that prevention would be better than cure. However, as every treatife on the difeafe defcribes its hiftory and regular progrefs from ftage to ftage, I fhall content myfelf with what is already faid on this part of the fubject, and fhall attempt a few ideas refpecting the method of cure.

The cure of Gout is not to be expected from a pill or a potion; charms and amulets will avail nothing; neither is it the work of one, or perhaps, in fome cafes, of feveral years, entirely to eradicate a difeafe which has been fo many in eftablifhing itfelf. Neverthelefs, much mifery may in the mean time be anticipated by comfort. Should, however, this great object be attainable on any terms, or in any period of time, he who would not willingly fubmit to the means muft know little of the fufferings it inflicts, and be totally unacquainted with the nature of a difeafe which grows with our years, and curtails or renders miferable the latter half of the life of thofe who are fubject to it. This object then is to be obtained by moderating the paroxyfms while prefent, and by removing their recurrence to a greater diftance by attention during their intervals. The firft of thefe intentions is chiefly to be effected by topical applications. That no irritation ought to be added to the fyftem during the paroxyfms of Gout is entirely agreed on among Phyficians: But it is alfo agreed, that any remedies which, without producing irritation, may tend to moderate the violence of inflammation, are admiffible; as violent inflammation flammation weakens the tone of the parts, and invites a more frequent return of the paroxyfms, which become more violent, and affect parts of greater confequence as the difeafe advances. Electricity has been found ferviceable, in fome cafes, during the wane of the paroxyfms, by taking fparks from the parts affected, the only way in which its application is admiffible. Great care and caution are, however, neceffary, not to excite the part beyond that degree which its ftate of collapfe at the time fpecifically demands. The application of cold alfo, at this period, under proper management, may be made to produce beneficial effects, by reftoring tone to the parts and vigour to the fyftem. But the inflammatory fpafm of the parts affected, during its greateft violence, is beft fubdued by a proper application of warmth, and that perhaps in the form of vapour, regularly and uniformly, for fome time, applied at a proper temperature. For this purpofe, the Air-pump Vapour Bath of Mr. Smith, mentioned in your Journals of April and July laft, is, beyond doubt, the means beft adapted for the application of it. Vapour, while it produces all the good effects of warm bathing, does not at the fame time debilitate and relax. According to my obfervations on its application in the above manner, it feems uniformly to produce tonic effects, which removing the preffure of the atmofphere at the fame time, (by making the parts take on a new action, and by powerfully promoting *that* of the abforbents) feems very much to aid. The capacity of the vafcular fyftem, and the propelling power of the *vis a tergo*, are at the fame time increafed. The violence of the pain, and of courfe the fyftematic fever, are abated, and the excitability fuffered to accumulate.

Large portions of the lithiat* of lime are alfo, during this application, frequently removed, and would, in every inftance, I am perfuaded, be prevented from forming by a timeous application of it. This fubftance, which ought to be depofited in the bones, but from the weaknefs of the veffels deftined to perform this office, is not fuffered to reach its deftination, collects in fuch quantities as to bolfter up the joints of the toes and ankles fo as to render them completely anchylofe. This matter, by an uniform action kept up on the parts for fome time, and frequently repeated by well-regulated applications of the machine, is in a great meafure removed, and limbs rendered

* Vid. Examen des Experiences & des Obfervations nouvelles de M. Pearfon fur les Concretions Urinaires, &c. d' l'homme par Mons. Fourcroy, Annales de Chimie, vol. xxvii.

dered ufeful, which would not be the cafe by any other means with which we are acquainted.

The paroxyfms are placed at a greater diftance, and thus the fecond intention is alfo powerfully aided by this application. The action of the difeafe is fufpended, while all the other remedies may be employed in turn. The principal one with this intention is a radical and fyftematic change of that mode of living which originally induced it. The miferable patient muft retrace his fteps; but this muft be done gradually, and with great caution. The exciting caufes muft be referred to, and their undue action as gradually diminifhed as it was induced. The advice of old Dr. Pitcairn to the Highland Chieftain, who had been in the habit of drinking a large goblet of whifkey every day, and who was defirous of leaving it off, ought to be attended to, which was as follows: "That he fhould melt as much wax every day into the goblet as would admit of the impreffion of his feal;" by which means the quantity of the whifkey was diminifhed daily, and the habit got rid of in the beft poffible manner.

The ingenious but unfortunate Dr. Brown purfued a different plan. Suppofing the enemy might be taken by a coup-de-main, he boldly pufhed as a remedy the concentrated force of the caufes of the difeafe. On this rock he fplit, and expiated his fate! a melancholy proof, that when great genius falls into errors, thofe errors are frequently not of a trivial nature. A proof alfo, that he who certainly furpaffed mankind in genius, either miftook his own doctrine, or, what is infinitely lefs to the credit of humanity, was not able to refift thofe habits, which never fail of producing the fame cataftrophe. Higher ftimulants than ufual may for a time give relief, but eventually produce greater debility; and the example of Dr. Brown ought to operate as a negative one for ever.

The deceitful, injurious, and mifchievous effects of the Portland powder, I fhould have confidered as unneceffary to have mentioned here, had not the ingenious and venerable Dr. Heberden declared his diffent from thofe who afcribe to its dangerous effects.*

Werlhoff†, a German, and Firft Phyfician to his late Ma-

* Pulvis Portlandicus præ fe fert æque adverfus utrumque prodeffe; neque negaverim hujufmodi medicamenta, fimul cum opio adhibito fic ut opus fuerit, fieri poffe præfentiffima antarthritica pariter et antirheumatica remedia.

† Sed ex nimio horum amaricantium ufu, fermentum ftomachi adeo debilitatum effe memini, ut nonnulli appetitum amiferint, cibos non concoxerint, mortem hinc potius quam fanitatem accelerarint; malique et infaufti remedii fævas dederint pœnas.——Werlhoff Cant. Med. p. 316.

jesty, for Hanover, condemns bitter remedies in the Gout, and is supported in the opinion by Murray*, the Gottingen Professor. Doctors Cullen, Darwin, and Falconer of Bath, strenuously support the sentiments of the latter, and adduce the most unequivocal proofs of their baneful effects. "In every instance," (says Dr. Cullen in his Practice of Physic, sec. 557) "which I have known of its exhibition for the length of time prescribed, the persons who had taken it were, indeed, free from any inflammatory affection of the joints, but they were afterwards affected with many symptoms of the Atonic Gout, and all, soon after finishing their course of medicine, have been attacked with Apoplexy, Asthma, or Dropsy, which proved fatal." In a later publication, he observes, "It is possible that several persons may have taken the Portland Powder and other bitters with seeming great advantage, but I have not had opportunity to know the sequel of the whole of such persons lives, so as to say positively how far, in any case, the cure continued steady for a life of some years after, or what accidents happened to their health. But I have had occasion to know, or to be exactly informed, of the fate of nine or ten persons who had taken this medicine for the time prescribed, which is two years. These persons had been liable for some years before to have a fit of regular or very painful inflammatory Gout, and particularly when they had completed the course prescribed, had never a regular fit, or any inflammation of the extremities for the rest of their lives. In no instance, however, that I have known, was the health of these persons tolerably entire. Soon after finishing the course of their medicine they became valetudinary in different shapes, and particularly were much afflicted with dyspeptic, and what are called nervous complaints, with lowness of spirits. In every one of them, before a year had passed, after finishing the course of the powders, some hydropic symptoms appeared, which gradually increasing in the form of an ascites, or hydrothorax, especially the latter, joined with anasarca, in less than two, or at most, three years, proved fatal. These accidents happening to persons of some rank, became very generally known in this country, and has prevented all such Experiments since.†.

---

* Ex pulvere Arthritico multi Apoplexiam, paralysin vel morbos acutos, senes precipuè, contraxerunt. Et in homine, quodam arthritis quidem inde sedata, sed respiratio difficilis, tussis sicca, morsque subitanea, successit, tuberculis pulmonum post mortem conspicuis.

Murray, vol. i. p. 355.

† Cullen's Mat. Med. vol. ii. p. 65, 66.

Were

Were it neceſſary to adduce further proof, Soranus among the antients might be mentioned, as bearing teſtimony that theſe remedies were then held in no better eſtimation than at preſent.

A ſteady adherence to an abſtemious and regular courſe of diet, conſiſting of milk, vegetables with an admixture of animal food, of the mildeſt kind, ſeems to be the only plan to be depended on for a radical cure of the gout.

The advice Dr. Darwin gave to one of his patients, applies beſt perhaps to the majority of caſes, which was as follows:

" Drink no malt liquor on any account. Let your beverage at dinner conſiſt of two glaſſes of wine diluted with three half pints of water. On no account drink any more wine or ſpirituous liquors in the courſe of the day; but if you want more liquid, take cream and water, or milk and water, or lemonade, with tea, coffee, chocolate. Uſe the warm bath twice a week for half an hour before going to bed, at the degree of heat moſt grateful to your ſenſations. Eat meat conſtantly at dinner, and with it any kind of tender vegetables you pleaſe. Keep the body open by two evacuations daily, if poſſible, without medicine; if not, take the ſize of a nutmeg of lenitive electuary occaſionally, or five grains of rhubarb every night. Uſe no violent exerciſe which may ſubject yourſelf to ſudden changes from heat to cold; but as much moderate exerciſe as may be without being fatigued or ſtarved with cold. Take ſome ſupper every night, a ſmall quantity of animal food is preferred; but if your palate refuſes this, take vegetable food, as fruit pie, or milk; ſomething ſhould be eaten, as it might be injurious to faſt too long.*

Dr. Cadogan has laid down ſome ſimple and judicious rules reſpecting regimen and management in a Treatiſe on this diſeaſe, which might be conſulted with advantage by thoſe labouring under it. Spirituous and fermented liquors, and fatigue of body and mind, ought equally to be avoided. Long faſting, night watching, and above all, the depreſſing paſſions, are extremely prejudicial. It is more from attention to thoſe circumſtances, and to judicious management with regard to them, than to any medicine either regularly preſcribed or empyrically recommended, that a cure is to be looked for; and notwithſtanding the boaſted powers of medicine and noſtrums, I am of opinion that little is to be done for the cure of this diſeaſe through the medium of the ſtomach, except by an uniform and gradual diminution of ſtimuli; and finally, a total abſtinence from ſpirituous

* Darwin's Zoonomia. Claſs, iv. 1, 2. Vol. ii. p, 457.

 and and fermented liquors. Neverthelefs, fhould the ftomach or head be attacked, or threatened with an attack, during the progrefs of cure, cordials and ftimulants are then not only admiffible, but ought to be adminiftered with fome degree of freedom. Madeira wine, with this double intention, is doubtlefs among the beft. To obviate coftivenefs, under thefe circumftances, nothing lefs warm than the tinctures of rhubarb or fenna ought to be adminiftered. That degree of excitement which the languid ftate of the fyftem requires ought to be duly fupplied by every poffible means. The application of heat is certainly among the moft powerful of thefe; and the beft mode of making it, *that* already alluded to, while the removal of the atmofpheric preffure from the extremities feems to have confiderable effect in determining the affection to them. The caufes and cure of this difeafe, to a certain extent, are the fame, and differ only in the proportions and manner in which they are applied. This feeming paradox is well expreffed by a Roman poet:

> "Balnea, Vina, Venus confumunt corpora noftra,
> Sed Vitam faciunt Balnea, Vina, Venus.",

Thus it feems, that while debauchery and inactivity are the fources from whence the difeafe fprings, temperance and activity never fail to prevent, and feldom to cure it, even after repeated attacks. Life is no longer defirable than whilft it can be enjoyed with fatisfaction and comfort. The firft rule towards acquiring this, is to avoid excefs in eating, drinking, and in mental exertion; to cultivate whatever promotes good humour, a chearful difpofition, and good fpirits. Regularity of hours, early rifing, a due proportion of fleep, pure air, wholefome food, warm cloathing, and moderate exercife, are of effential importance: But to expect a radical cure of the Gout without regard to thefe; or, while the fame plan which gave rife to it, is purfued, would be as ridiculous as though a perfon who had been cured of a leg broken by jumping down precipices, fhould expect that his having been cured of it once, fhould enfure him againft the like accident in future, though he fhould continue to run the fame rifks.

*Sept.* 13, 1802.

